# MicroRNAs in the Progress of Diabetic Nephropathy: A Systematic Review and Meta-Analysis

**DOI:** 10.1155/2019/3513179

**Published:** 2019-03-07

**Authors:** Li-ping Wang, Yu-zhen Gao, Bin Song, Guo Yu, Hui Chen, Zhen-wen Zhang, Cai-feng Yan, Yun-long Pan, Xiao-yan Yu

**Affiliations:** ^1^Department of Biobank, Clinical Medical College, Yangzhou University, Northern Jiangsu People's Hospital, Yangzhou, Jiangsu, China; ^2^Department of Endocrinology, Clinical Medical College, Yangzhou University, Northern Jiangsu People's Hospital, Yangzhou, Jiangsu, China; ^3^Department of Experimental Pharmacology and Toxicology, School of Pharmacy, Jilin University, Changchun, China

## Abstract

**Background:**

We conducted a systematic review and meta-analysis of existing literature to evaluate the different outcomes of microRNAs (miRNAs) in diabetic nephropathy (DN), including urinary albumin excretion rates, urinary albumin creatinine rates, glomerular filtration rate, HbAc1, and creatinine.

**Methods:**

Electronic databases including PUBMED, MEDLINE, and EMBASE were searched for eligible publications to July 2018. The following comparisons between treatment groups were included: normal group versus DN group; control group versus micro/macroalbuminuria group.

**Results:**

Twelve eligible studies that included 2500 participants were finally recruited in this meta-analysis. Fifteen miRNAs (miRNA-21, miRNA-181b, miRNA-194, miRNA-30, miRNA-215, and others) were upregulated whereas seven miRNAs (miRNA-26a, miRNA-126, miRNA-424, miRNA-574-3p, miR-223, miR-155, and miR-192) were downregulated in the DN group compared with control groups. The miR-133b, miR-342, miR-30, miR-192, miR-194, and miR-215 were significantly correlated in urinary albumin excretion rates (r=0.33, 95% CI= 0.26-0.39). miR-192, miR-217, miR-15b, miR-34a, and miR-636 were correlated with urinary albumin creatinine rates (r=0.69; 95% CI=0.12-0.92), while miR-133b, miR-345, miR-33, miR-326, miR-574-3p, miR-126, miR-217, miR-15b, miR-34a, and miR-636 were significantly correlated with HbAc1 (r =0.23, 95% CI = 0.15-0.31). There were twelve miRNAs that were closely related to the glomerular filtration rate (r=0.28, 95% CI =0.21-0.34). Creatinine (r=0.33, 95% CI = 0.22-0.40) was significantly different between normal and DN groups.

**Conclusions:**

The meta-analysis acquired the correlations between miRNAs and outcomes including UAER, UACR, eGFR, HbAc1, and creatinine in DN. It suggested that miRNAs may participate in the pathogenesis of DN process.

## 1. Introduction

Diabetic nephropathy (DN) is one of the most serious and prevalent complications that can lead to death in diabetic patients, as well as being a major contributing factor to end-stage renal disease (ESRD) [1]. The stages of DN are defined as incipient, manifest, and advanced, with DN usually developing over a period of years [2]. The earliest clinical indication of DN is the appearance of abnormally low levels of albumin in the urine (microalbuminuria) [3]. The onset of microalbuminuria leads to macroalbuminuria, and the latter is followed by deterioration of renal function with a progressive decline in the glomerular filtration rate (GFR), which eventually leads to ESRD [4]. Although microalbuminuria is considered as the gold standard for the diagnosis of DN, renal dysfunction can also be identified through other variables including urinary albumin excretion rates, urinary albumin creatinine rates, glomerular filtration rate, HbAc1, and creatinine. Identifying patients in the early stage of DN is an important step for effective management and treatment [5].

MicroRNAs (miRNAs) are 19-25 nucleotide (nt) regulatory RNAs that suppress the translation and stability of mRNA through imperfect base pairing in the 30 untranslated region of its mRNA targets [6]. These are widely found in plants, nematode worms, and human cells. miRNA can bind to specific sites within the 3′UTR of its target mRNAs with incomplete complementary to inhibit protein synthesis [7]. The role of miRNAs in the occurrence and development of various diseases has become a hot topic in the field of life sciences. Recently, some studies have shown that miRNAs influence the expression of gene regulation to participate in the pathogenesis of DN processes and play an important role in the pathogenesis of DN [7]. This paper used the method of evidence-based medicine to evaluate the standard related literature and perform a meta-analysis and comparative analysis, in order to objectively ascertain the involvement of miRNAs in the pathogenesis of DN and how related indicators may be affected.

## 2. Materials and Methods

### 2.1. Data Sources and Search Strategy

Electronic databases including PUBMED, MEDLINE, and EMBASE were searched to identify eligible studies to July 2018 inclusive, using the following key words: “MicroRNA”, “miRNAs”, “Micro RNA”, “RNA, Micro”, or “miRNA”; “DN”, “Diabetic Nephropathy”, “Nephropathies, Diabetic”, “Kidney Disease, Diabetic”, “kidney”, “renal”, or “nephrodium”; “human”, “patients”, or “people”. Furthermore, the reference list of every article was retrieved and reviews were manually searched to identify additional eligible studies.

### 2.2. Eligibility Criteria

Study inclusion criteria were as follows: (1) diabetic nephropathy was defined according to the American Diabetic Association when using the random collection technique, where normal urinary albumin creatinine ratio (UACR) was defined as<30 mg/g creatinine, microalbuminuria (MA) was defined as 30-299 mg/g creatinine, and macroalbuminuria (MAA) was ≥300 mg/g creatinine; (2) investigating the association between miRNA expression and DN outcomes (UAER, UACR, eGFR, HbAc1, and creatinine) and (3) the utilized miRNA detection methods were clearly defined. Studies were excluded if they were case reports, letters, conference records or review, and animal studies.

### 2.3. Data Collection

Eligibility evaluation and data abstraction were independently analyzed by 2 investigators (L.W, Y.G) according to the guidelines of the Meta-analysis of Observational Studies in Epidemiology group and the discrepancies were adjudicated by consensus. For each study, the following data were extracted: first author; year of publication; miRNAs; country; methods; included case number (DN/Normal); samples; outcome measures (abnormal miRNAs expression; the clinical data changes by miRNAs; the correlation of miRNAs expression; target of miRNA; signaling pathway of miRNAs). The r coefficient values among miRNAs and other clinical indices of the DN groups were also extracted for the meta-analysis.

### 2.4. Statistical Analyses

All the mean and absolute R correlation coefficient values were merged for meta-analysis using R program (version 3.4, R Inc., USA). The package of “meta” was adopted in the meta-analysis. The main program statements were “metacont” and “metacor” in the process of the meta-analysis. Standardized Mean Difference (SMD) and 95% confidence intervals (95% CIs) were summarized using the random-effects model or fixed-effect model according to heterogeneity. Spearman r was translated into Pearson r, while “ZCOR” heterogeneity between studies was assessed by the chi-squared test and I^2^ with* P *<0.05 indicating significant heterogeneity: I^2^ results interpretation: I^2^ <25%: low heterogeneity; I^2^=25-50%: moderate heterogeneity; I^2^>50%: a high degree of heterogeneity.

## 3. Results

**Figure 1:**
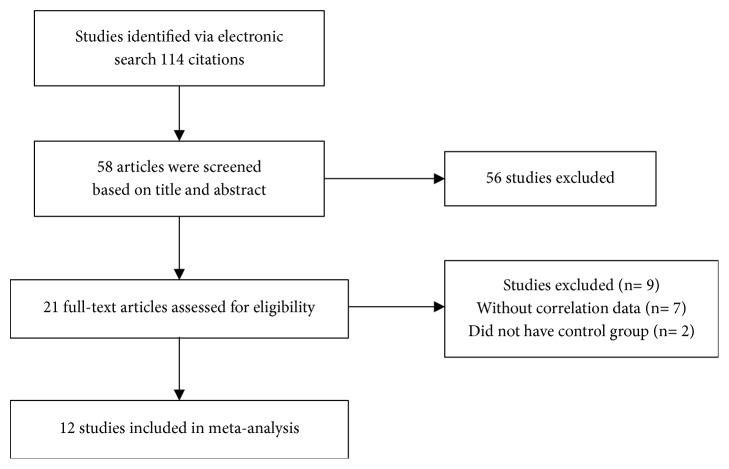
The flow diagram.

The electronic search retrieved a total of 114 citations. Of these, 56 articles were excluded on the basis of title and abstract; 21 articles were assessed fully for eligibility. A total of 9 articles were excluded for the following reasons: (a) correlation results without data (n=7) and (b) absence of a control group (n=2). Therefore, 12 studies were finally included in this meta-analysis (Figure 1). The characteristics of the included studies are listed in Table 1 [8–19].

**Figure 2:**
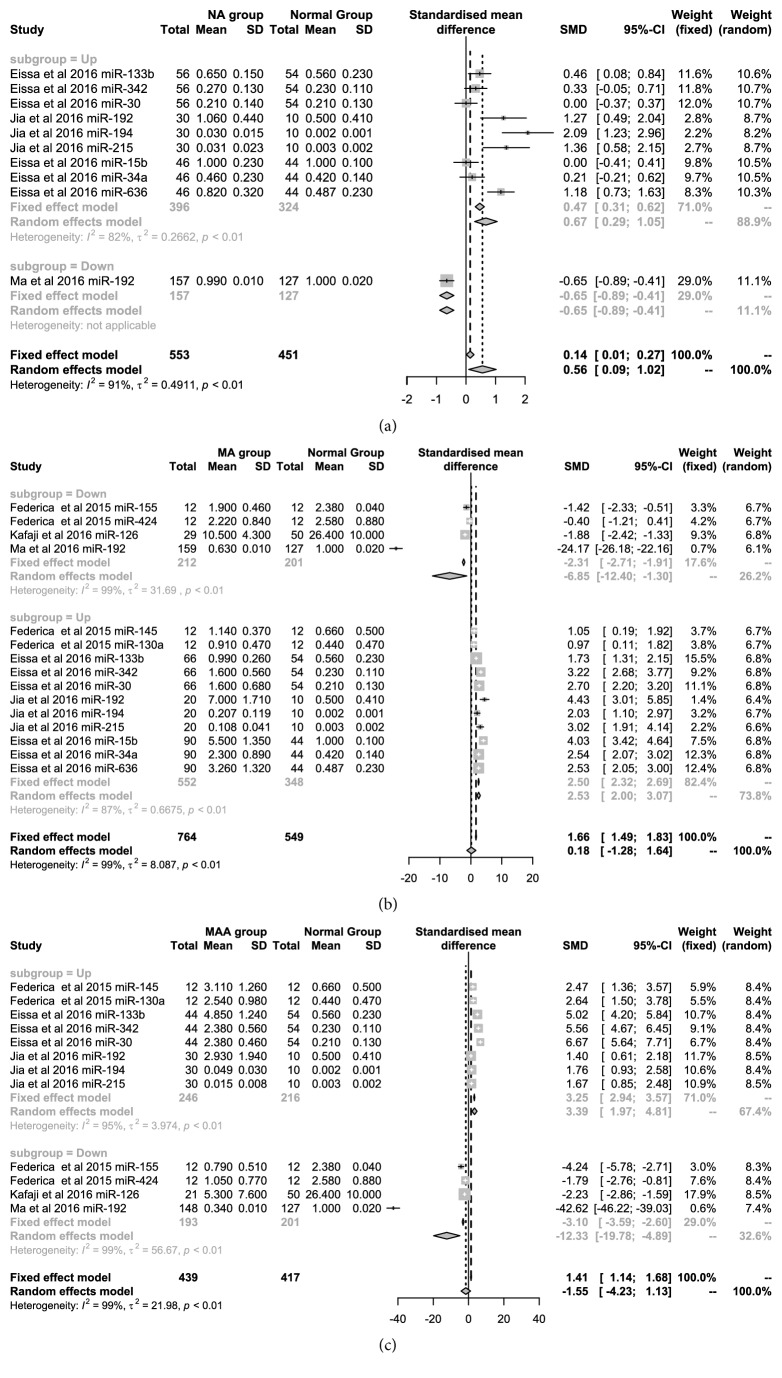
Forest plots for mean values of miRNAs in the patients with DN.

In the 12 articles, the expression value of different types of miRNAs we analyzed, which had been researched among the NA, MA, MAA, and normal groups. Moreover, we also conducted subgroup analysis for miRNAs that were up- and downregulated in the meta-analysis.  Figure 2(a) showed the difference in miRNA expression between the NA and normal groups in five articles. The upregulated miRNAs were miR-133b, miR-342, miR-30, miR-192, miR-194, miR-215, miR-15b, miR-34a, and miR-636. We further adopted the Random Effect Model (SMD=0.67, 95%* CI*: 0.29-1.06) due to high heterogeneity (*I*^*2*^=82%,* P*<0.01).  Figure 2(b) showed that there were 4 upregulated and 11 downregulated MA-related miRNAs from five articles, which were pooled using the random-effects model with 99% heterogeneity. The SMD values were 2.53 (95%* CI*: 2.00-3.07) and -6.86 (95%* CI*: -12.40-1.30) for upregulated and downregulated miRNAs, respectively. In Figure 2(c), the expression of 8 upregulated miRNAs from three articles was pooled using the random-effects model with high heterogeneity (*I*^*2*^=82%-99%) and the SMD was 3.39 (95%* CI*:1.97-4.81) between the MAA and normal groups. The SMD of four downregulated miRNAs was 12.33 (95%* CI*: -19.78--4.89) between two groups from three articles.

### 3.1. Relationship of miRNAs with eGFR

**Figure 3:**
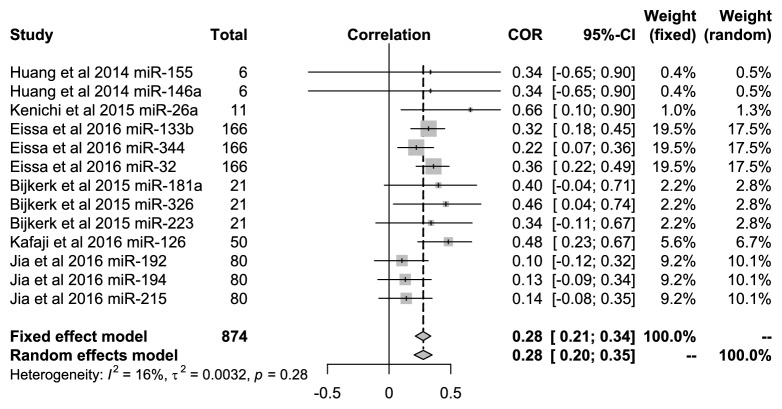
Forest plots for the relationships of miRNAs with the eGFR in DN patients.

In all included studies, Koga et al. [16] showed that the related expression of miR-26a was significantly related to eGFR, and Eissa et al. [12] showed that the miRNAs were miR-133b, miR-344, and miR-32. In addition, there were significant correlations among miR-326, miR-126, and eGFR in the research by Bijkerk et al. [17] and Al-Kafaji et al. [14] (*P*<0.05). All 13 types of miRNAs were included to evaluate the relationship with eGFR in DN patients in 5 articles.  Figure 3 showed that the pooled correlation value was 0.28 (95%* CI*: 0.21-0.34) under the fixed-effect model with low heterogeneity (*I*^*2*^=16%,* P*=0.28).

### 3.2. Relationship of miRNAs with HbAc1

**Figure 4:**
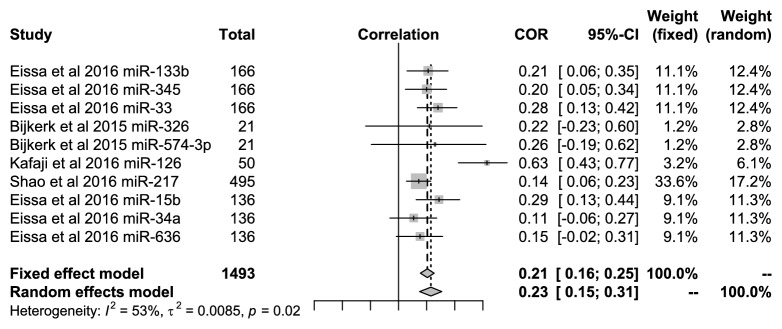
Forest plots for the relationships of* miRNAs *with the HbAc1 in DN patients.

Similarly, 10 miRNAs were included to evaluate the relationships with HbAc1 in DN patients. Among the studies, miR-133b, miR-345, miR-33, miR-126, miR-217, and miR-15b were significantly related to HbAc1 in DN patients in the studies by Eissa et al. [12], Bijkerk et al. [17], and Al-Kafaji et al. [14] (*P*<0.05).  Figure 4 showed that the pooled correlation value was 0.23 (95%* CI*: 0.15-0.31) under the random-effects model with high heterogeneity (*I*^*2*^=53%,* P*=0.02).

### 3.3. Relationship of miRNAs with Creatinine

**Figure 5:**
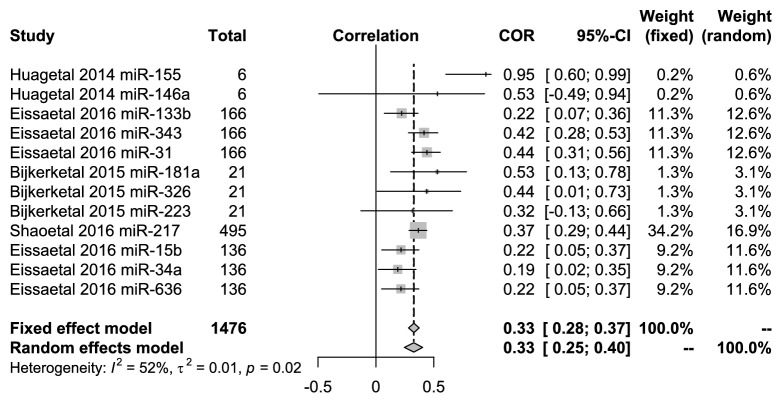
Forest plots for the relationships of* miRNAs *with the serum creatinine in DN patients.

There were 12 miRNAs in 5 articles, which were evaluated for their relationship with creatinine values in DN patients. miR-146a and miR-223 were not clearly correlated with the level of creatinine, respectively, in the studies of Bijkerk et al. [17] and Huang et al. [18]. However, the other 10 miRNAs (miR-155, miR-133b, miR-343, miR-31, miR-181a, miR-326, miR-217, miR-15b, miR-34a, and miR-636) were significantly correlated.  Figure 5 showed that the pooled correlation value was 0.33 (95%* CI*: 0.25-0.40) under the random-effects model with high heterogeneity (*I*^2^ =52%,* P*=0.02).

### 3.4. The relationship of UAER and UACR with miRNAs

**Figure 6:**
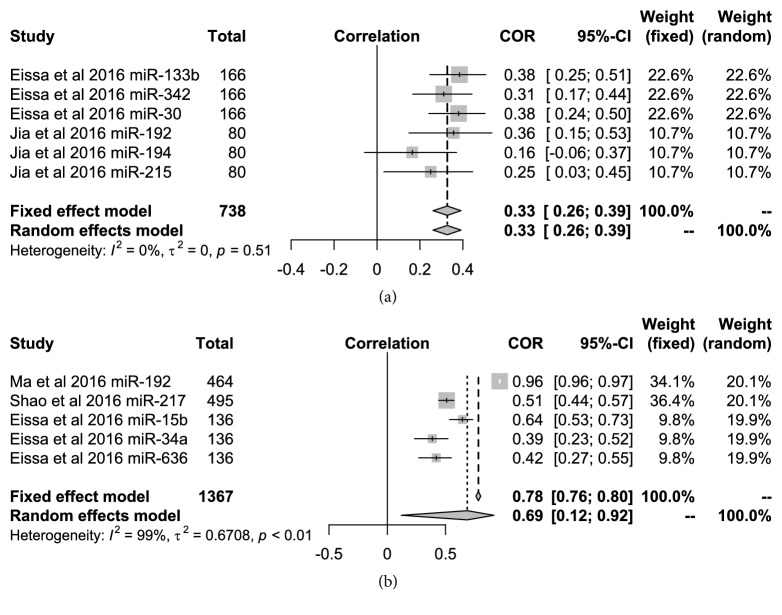
Forest plots for the relationships of* miRNAs *with the UACR and UAER in the DN patient.

There were five miRNAs that appeared to be significantly related to UACR in the DN patients (miR-192, miR-217, miR-15b, miR-34a, and miR-636) in the included articles. However, the study by Jia et al. [11] showed that miR-194 was not related to UAER. The five following categories of miRNAs (miR-133b, miR-342, miR-30, miR-192, and miR-215) were related with UAER. Figures 6(a) and 6(b), respectively, shows that the relationship of UAER and UACR with miRNAs in the DN patients. The pooled r coefficient was 0.33 (95%* CI*: 0.26-0.39) by the fixed-effect model with low heterogeneity (*I*^*2*^=0,* P*=0.51) between UAER and 6 types of miRNAs in two articles. However, the heterogeneity was very high at 99% (*P*<0.01) when the correlation between UACR and the 5 types of miRNAs were pooled from the three articles.

## 4. Discussion

DN is a common complication of diabetes mellitus (DM), which can eventually lead to end-stage renal disease (ESRD) [20]. This can seriously affect health and lowers the quality of life in these patients. The accumulation of the extramural matrix in the glomerular membrane and the basement membrane of the cell membrane are pathological features of DN, eventually leading to glomerular fibrosis. The main characteristics of DN are continuous proteinuria, renal impairment, and a high incidence of cardiovascular disease and mortality [21]. At present, the early diagnosis and monitoring of DN mainly rely on the detection of microalbuminuria, but the level of it often cannot accurately reflect the specific situation of DN. Besides, renal biopsy can diagnose and judge disease progression, but it cannot be widely carried out due to its high risk of invasion and complications. Therefore, it is urgent for us to use markers with higher specificity and sensitivity for DN diagnosis.

MiRNAs can interfere with the protein synthesis, degrade mRNA, or inhibit protein translation to regulate cell growth, differentiation, apoptosis, and proliferation. MiRNAs have become the focus of important research studies in recent years. Increasing evidence indicates that miRNAs can affect the progress of DN by regulating various signal pathways in the pathogenesis of DN, such as TGF-*β*, Akt, NF-B, etc. MiRNAs can also be highly specific in the body fluids of patients, and its degree of change is often more sensitive than the currently commonly used clinical indicators (Such as urine protein). Therefore, miRNAs can be used as an emerging biomarker to assist in the early diagnosis of DN and the evaluation of disease outcome.

From the 12 articles, a total of 2500 patients were included in this systematic review and meta-analysis. The data show that miRNAs may influence DN progression and pathogenesis through the TGF-*β*/Smad7 signaling pathway. McClelland et al. [15] found that the expression of miR-21 was significantly upregulated in renal biopsy tissues of DN patients with rapid disease progression and high degree of fibrosis. When the Smad3 and Akt pathways of proximal renal tubular epithelial cells (PTCs) were blocked, the TGF-*β*1 and PTCs collagen were decreased under the treatment of miR-21, suggesting that miR-21 is involved in the regulation of collagen and fibronectin and other sclerosis-promoting effects of TGF-*β*1-mediated Smad3 and Akt signaling pathways. Koga et al. [16] identified that miR-26a, which targets the CTGF gene, is a potent suppressor of TGF-*β*/Smad signaling in cultured human podocytes. Overactivity of TGF-*β*/Smad signaling stimulates the production of extracellular matrix and plays an important role in the pathogenesis of DN. We divided the patients into four groups (normal, NA, MA, and MAA groups) to analysis whether there were miRNA differences. Compared with the normal group, there were nine upregulated and one downregulated miRNAs in the NA group and 4 upregulated and 11 downregulated miRNAs in MA group. These observations were confirmed using 5 articles that were pooled by using the random-effects model with 99% heterogeneity. The SMD values were 2.53 (95% CI: 2.00-3.07) and -6.86 (95% CI: -12.40--1.30) between the MAA group and normal group, respectively. There were four downregulated miRNAs identified with a SMD value of 12.33 (95% CI: -19.78--4.89) from three articles. It has been suggested that miRNAs may participate in the pathogenesis process at different periods of the DN process.

The urinary albumin excretion rate (UAER) test can evaluate the degree of renal and vascular endothelial damage in DN patients [22]. Positive and reliable treatment for early DN patients can improve and advance prognosis. Chao et al. [23] showed that UAER is widely accepted as one of the criteria for the diagnosis and clinical grading of DN, while MA has been recommended as the first clinical sign of DN. In this study, we found that the UACR was not affected by other factors and is more suitable for monitoring the UAER situation. The changes in UACR occur earlier than blood urea nitrogen (BUN) and creatinine abnormalities, which are sensitive indices for early diagnosis of diabetic renal injury [24]. Zhanget al. [25] found that overexpression of miR-21 inhibited proliferation of mesangial cells and decreased the 24-h UAER in diabetic db/db mice. Our study, respectively, analyzed 6 and 5 types of miRNAs between UAER and UACR in DN patients. The correlation was p = 0.51 and* p* < 0.01.

Glomerular filtration rate (eGFR) is an important functional index based on diagnosis and staging of chronic renal disease (CKD) [26]. In 2016, Pan longitudinally analyzed eGFR for prediction of ERSD. The ROC curves for eGFRcre-cys (AUC=0.86±0.03) over 24 months were increased compared with the ROC curves for eGFRcre and eGFRcys (*p*<0.05), which suggested that the eGFR cre-cys equation may be more precise and sensitive for predicting renal outcome in T2DN patients [27]. In 2017, it was reported that AG1478 can act as a specific inhibitor of eGFR, and can improve ROS accumulation and ER stress by inhibition of eGFR and AKT activation, preventing kidney injury caused by diabetes. Therefore, the EGFR/AKT/ROS/ER stress pathway is blocked and appears to prevent renal injury caused by DN [28]. These results indicate that eGFR plays a critical role in the development of DN, and inhibiting eGFR may be a potential treatment strategy for DN. In non-small-cell lung cancer (NSCLC), eGFR acts as a direct target of miRNA, and siRNA knockdown of EGFR can inhibit cell proliferation, promote apoptosis, and arrest cell-cycle progression. The findings suggest that miRNA inhibits NSCLC tumor growth and metastasis through targeting of EGFR [29]. In our study, there were 13 types of differentially expressed miRNAs in two groups and the relationship of miRNAs with eGFR was p=0.28. Glycosylated hemoglobin (HbAc1) is the product of the combination of hemoglobin and blood glucose in red blood cells in human blood, and this parameter usually reflects the patient's blood glucose control over a period of 8-12 weeks. Long-term HbAc1 variability predicts deterioration of chronic kidney disease in type 2 diabetic patients with preserved kidney function [30]. Shao et al. [8] found that type 2 DN group HbAc1 levels were significantly higher than a normal group and a type 2 diabetes unincorporated nephropathy group. Further clarification of HbAc1 levels in type 2 diabetes patients may improve the effective diagnosis of DN patients. Chinese studies of serum samples suggest that microRNA-217 may be associated with the development of proteinuria in type 2 diabetes patients and may be involved in the development of DN by promoting chronic inflammation, renal fibrosis, and angiogenesis. This particular miRNA was positively correlated with diabetes mellitus duration, a homeostasis model assessment for insulin resistance, glycated hemoglobin, serum creatinine, and other outcomes [31]. Our study analyzed 10 types of miRNA (miR-133b, miR-345, miR-33, miR-326, miR-574-3p, miR-126, miR-217, miR-15b, miR-34a, and miR-636) to evaluate the relationships with HbAc1 in DN patients in 5 articles. They appeared to be significantly associated (p<0.05).

Creatinine is a small molecule that can be filtered through the glomeruli and is rarely absorbed in the renal tubule. The daily level of creatinine produced by the body is excreted almost entirely in the urine and is not affected by the urine volume. Clinical detection of blood creatinine is one of the main methods to understand renal function. Cao et al. [32] suggest that the serum creatinine levels of patients with DN are significantly higher than those in the control group. Spearman correlation analysis found that DN patients had a correlation with serum creatinine levels. Serum creatinine levels can help determine the prognosis of DN patients but the clinical sensitivity of this parameter is not very high. Tabriz and colleagues through assessed plasma samples for circulating miRNAs and found that expression levels of miR-21 in plasma correlated with creatinine (*r* =-0.432,* P* =0.03) [33]. In our study, we selected 12 types of miRNAs (miR-155, miR-146a, miR-133b, miR-343, miR-31, miR-181a, miR-326, miR-223, miR-217, miR-15b, miR-34a, and miR-636) from 5 articles and correlation analysis found that they were closely related (*P*=0.02).

In our study, we identified 14 miRNAs that were obviously changed in this study and showed that the expression of these miRNAs was associated with clinical indicators of DN. Among them, miR-21 and miR-133b are two misregulated miRNAs which have been extensively studied in DN. Global expression profiling revealed miR-21 to be among the most highly regulated microRNAs in kidneys of mice with DN [34]. miR-21 correlated with tubulointerstitial injury in kidney biopsies of diabetic patients and showed a specific enrichment in glomerular cells use situ PCR analysis. MiR -21 can adjust the mesangial expansion, interstitial fibrosis, macrophage infiltration, podocyte loss, albuminuria, and fibrotic- and inflammatory gene expression related to DN [35]. Eissa et al. [12] showed that miR-133b may be an important biomarker for the prediction of early DN.

In conclusion, the meta-analysis revealed the correlations between miRNAs and clinical indicators of DN. However, the number of included articles was limited; the above conclusions still need to be verified by high-quality studies. We will focus on the specific pathogenesis of miRNAs involved in the occurrence and development of DN for further research, so as to provide new ideas for the early diagnosis and treatment of DN.

## Figures and Tables

**Table 1:** Main characteristics of the reports included in the study; expression of miRNAs in DN groups.

Author	Year	Country	Methods	Samples	Case Number				MiRNAs	Expression
					N	NA	MA	MAA		
McClelland et al.	2015	Australia	qRT-PCR	FFPE	35	8			miR-21	up
Huang et al.	2014	China	qRT-PCR	biopsy	3	6			miR-155,miR-146a	up
Koga et al.	2015	Japan	qRT-PCR	biopsy				11	miR-26a	down
Eissa et al.	2016	Egypt	qRT-PCR	urinary exosomes	54	56	66	44	miR-133b,miR-342,miR-30	up
Bijkerk et al.	2015	Netherlands	qRT-PCR	plasma	19	21			miR-181a,miR-326	up
									miR-223,miR-574-3p	down
Federica	2015	Italy	qRT-PCR	urinary exosomes			12	12	miR-145,miR-130a	up
									miR-155,miR-424	down
Kafaji et al.	2016	Bahrain	qRT-PCR	whole blood	50		29	21	miR-126	down
Ma et al.	2016	China	qRT-PCR	serum	127	157	159	148	miR-192	down
Jia et al.	2016	China	qRT-PCR	urinary	10	30	20	30	miR-192,miR-194,miR-215	up
Barutta et al.	2016	Italy	qRT-PCR	serum	143		74	105	miR-126	down
Shao et al.	2016	China	qRT-PCR	serum	195	186	169	140	miR-217	up
Eissa et al.	2016	Egypt	qRT-PCR	urinary exosomes	44	46	90		miR-15b,miR-34a,miR-636	up
